# Knowledge and Risk Perceptions of Chronic Kidney Disease Risk Factors among Women of Childbearing Age in Lagos State, Nigeria: From a Health Demography Approach

**DOI:** 10.1155/2022/5511555

**Published:** 2022-05-19

**Authors:** Monica Ewomazino Akokuwebe, Erhabor Sunday Idemudia

**Affiliations:** North-West University Faculty of the Humanities, North West University, Mafikeng, South Africa

## Abstract

**Background:**

Kidney disease (KD), also known as chronic kidney disease (CKD), is a long-term underrecognized public health concern and one of the eight leading causes of death in women. Despite that, little is known about women's knowledge, perceived risk, and perceptions of CKD risk factors. In this study, we assessed knowledge, perceived risk, and perceptions of CKD risk factors among women of childbearing age in Lagos State, Nigeria.

**Methods:**

Administering a pretested and structured questionnaire among 825 women aged 15–49 years, we conducted a cross-sectional descriptive study to evaluate knowledge, self-reported CKD risk factors, and risk perception among women of childbearing age in urban and semiurban communities in Lagos State, Nigeria. We used descriptive (mean, frequencies, and percentages) and bivariate statistics (chi-square) to assess sociodemographic factors influencing knowledge and perceptions of CKD risk factors. Binary and multinomial logistic regressions were further employed to assess risk perceptions of CKD factors associated with knowledge.

**Results:**

Five hundred and forty (65.5%) out of 825 women reported being knowledgeable of CKD risk factors with majority of the younger adult women (15–29 years) having good knowledge than other age cohorts, with a mean age of 33.5 ± 11.5 years. The women's knowledge of CKD was found to be significantly associated with independent and dependent risk factors (*p* < 0.05). The major self-reported independent CKD risk factors were misuse of analgesics (NSAIDs) (OR = 1.20; *p* < 0.05), herbal drinks (OR = 2.30; *p* < 0.05), and herbal supplements (OR = 1.37; *p* < 0.05), while self-reported dependent CKD risk factors were hypertension (OR = 2.14; *p* < 0.05), family history of KD ailments (OR = 1.30; *p* < 0.05), and high cholesterol (OR = 1.44; *p* < 0.05). Similarly, majority of the women had low perceived CKD risk (54.8%), while women with CKD risk factors (independent and dependent) view themselves at decreased perceived risk for the disease compared to those who are not associated with CKD risk factors (*p* < 0.05). Also, findings revealed that women had poor perception of risk factors associated with CKD. The multivariate analysis of perceived risk showed that demographic factors (younger aged adults, high education, and high income), independent risk factors of CKD (misuse of NASAIDs and excessive use of herbal drink and herbal supplement), and dependent risk factors (hypertension and family history of KD ailments) were significantly associated with knowledge of CKD (*p* < 0.05).

**Conclusion:**

Our study reveals high knowledge of CKD risk factors but low perceived risk and poor perception of the link between CKD risk factors and its ailments. Given this, there is a call for urgent measures to create sensitization and provide public CKD behavioural health interventions as well as easy communication strategies for women to secure better access to awareness intervention programmes and healthcare services.

## 1. Background

Kidney disease (KD), classified into two—acute (occurring within a very short period, usually within days or a week) and chronic (arising with insidious onset and takes months or years to develop) kidney diseases (CKD)—is an underrecognized public health concern that causes more deaths than some other chronic conditions. CKD typically means one's kidneys are damaged which causes a loss of function, gradually resulting in kidney failure. Increasing incidence and prevalence of CKD is often associated with persons not having any symptoms in the early stages of the disease. However, later signs/symptoms are often accompanied by late presentation and limited access to replacement therapy owing to its unaffordability by affected persons. Its burden appears to be more marked in sub-Saharan Africa [1] and is a major cause of death in women worldwide, with estimated 600,000 deaths occurring each year [2–4]. Thus, CKD poses a high risk of morbidity and psychological distress to victims and their families as well as an enormous burden on health financing, especially among those without health insurance coverage [3, 4]. Most importantly, kidney treatment such as dialysis and renal replacement therapy are not included in health insurance plans in some countries, especially in developing nations [4, 5].

Recent demographical studies on CKD have illustrated that gender plays a significant role in predicting the likelihood of reaching end-stage CKD (GFR<15 mL/min) [4–6]. Medical and public health specialists have documented that woman are more susceptible to CKD than men, although men have shown higher odds of chronic kidney failure (CKF) sooner than women [7, 8]. Other nonmedical experts such as social scientists and health demographers have indicated “being male” as an important demographic variable used as a proxy for risk factor to arrive at end-stage CKD [9, 10]. This is because the differences in hormone levels, such as greater testosterone levels in men, may cause loss of kidney function [11, 12]; then again, men's kidneys may perhaps are not being protected by the hormone estrogen, which is higher in women up to menopause [11]. Generally, men have been considered to have more unhealthy lifestyles than women; and most especially, men undergo dialysis or kidney replacement at a younger age than women [2, 3, 11, 12]. Conversely, there is a dearth of feasibility studies that have indicated the possibility of females undergoing dialysis or kidney transplant at a younger age [11–13].

Consequently, in Nigeria, medical professionals have raised concern over the rising number of cases of CKD and the disease burden among women of childbearing age [14, 15]. Empirical studies have shown that 17,000 new cases of CKD are diagnosed annually. Besides, about thirty-eight million people suffer from various stages of the disease [14–16]. Often, women exhibit CKD symptoms at an advanced stage, especially when confronted with pregnancy-related complications such as preeclampsia and other maternal diseases; many of these women already have kidney damage [14–19]. This is a result of women not undergoing regular medical check-ups and with no health insurance cover, as most times, there are no prior signs/symptoms of the disease [3, 12, 15]. The cost of CKD treatment and renal replacement therapy in Nigeria is also high; thus, many patients cannot afford them [9, 10, 20]. Unfortunately, the Nigerian government in the National Health Insurance Scheme (NHIS) does not prioritize treatment of CKD. Thus, adequate knowledge of CKD risk factors will increase risk perception and readiness for lifestyle modifications as well as medical screening for early diagnosis. This would prompt early treatment and reduce deaths and high healthcare expenses [14, 15].

In Nigeria, community-based studies on knowledge and risk perceptions of CKD among women of childbearing age are few. Largely, previous studies focused on clinical symptoms of CKD among women [3, 15], neglecting their prior knowledge and risk perceptions of CKD as important factors in early diagnosis and treatments. Consequently, this study focuses on this strategy and promotes CKD awareness design, prevention, and screening services by targeting women of childbearing age using multiple media facilities. Based on this background, this community-based cross-sectional survey fills an important knowledge gap in the literature by examining knowledge and risk perceptions of KD development among women of childbearing age. In the year 2018, Lagos State resident nephrologists raised apprehension about the increasing number of cases of KD among women during the prenatal period [15]; thus, Lagos State was selected for this study.

## 2. Methods

### 2.1. Study Setting

For our study, data were collected from three senatorial districts in Lagos State, located in the southwestern region of Nigeria. Lagos State comprises twenty Local Government Areas (LGAs), grouped into three zones known as senatorial districts: Lagos Central, Lagos West, and Lagos East [21]. The Lagos Central senatorial district has five urban LGAs (Lagos Island, Lagos Mainland, Surulere, Apapa, and Eti-Osa). Lagos West senatorial district has ten urban LGAs (Agege, Ifako-Ijaiye, Alimosho, Badagry, Ojo, Ajeromi-Ifelodun, Amuwo-Odofin, Oshodi/Isolo, Ikeja, and Mush-in). Then, the Lagos East senatorial district consists of five semiurban LGAs (Shomolu, Kosofe, Epe, Ibeju-Lekki, and Ikorodu) [21]. Urban communities are centered in Lagos Central and Lagos West, while semiurban communities are found within the Lagos East senatorial district. Lagos State is also called the world's next “mega-city” due to its increasing urban population and economic development. It has an estimated population of 25 million with an increasing poverty rate.

In 2019, the Oxford Poverty and Human Development Initiative (OPHI) reported that 20% of the Lagos population is vulnerable to poverty. In addition, the intensity of economic deprivation in Lagos State stands at 41.1%, and several Nigeria citizens are living below the national poverty line of 69% [22]. The Lagos State health system exhibits medical diversity in Nigeria, which harmonizes public, private, and traditional medicine facilities [23]. However, Lagos State Monitoring and Accreditation Agency for healthcare facilities reported that the state government is putting in place policies that will bring quality healthcare to all residents of Lagos State. Still, the public healthcare facilities are not well stocked, not well managed, and have staff shortages. Equally, many of the facilities are run down [23]. Women do not have access to adequate healthcare, as many public hospitals are substandard and private hospitals are unaffordable [23]. Public healthcare provides services at the primary, secondary, and tertiary levels, and women receive antenatal and postnatal care from both primary and secondary healthcare.

### 2.2. Sampling

Data were collected from a household survey of 1,850 households conducted in September 2018–January 2019. It was a baseline survey for upcoming kidney health interventions. The purpose of the survey was to sensitize women within the communities in the senatorial districts where the interventions and health facilities were presumed to be initiated, about kidney risk factors and their ailments as well as referring persons with kidney disease symptoms. The justification for purposive sampling was determined by the urgent needs of the upcoming health intervention and sensitization programmes for women of childbearing age, especially those with high-risk factors.

**Figure 1:**
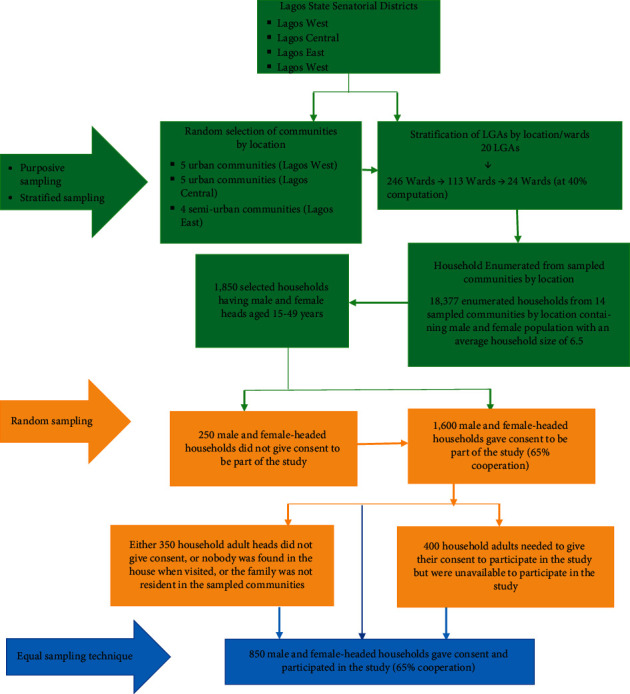
Flowchart showing sampling techniques used to select survey households and the selection of the household heads that participated in the study.

Multistage, stratified, and equal sampling techniques were applied to identify the households to be included in the survey. A mixture of random and purposive selection techniques were applied at each stage of sampling (Figure 1). First, three senatorial districts by urban and semiurban stratification were purposively selected in the survey: Lagos West and Lagos Central (urban) as well as Lagos East (semiurban). These three senatorial districts were selected because the Lagos State Ministry of Health has proposed a health intervention coverage scheme within the communities in these senatorial districts for easier dissemination of health information on kidney disease and its risk factors among the female population.

The three senatorial districts have twenty local government areas (LGAs): Agege, Ajeromi-Ifelodun, Alimosho, Amuwo-Odofin, Apapa, Badagry, Kosofe, Mushin, Oshodi Isolo, Ojo, Ikorodu, Surulere, Ifako-Ijaiye, Shomolu, Lagos Mainland, Ikeja, Eti-Osa, Lagos Island, Epe, and Ibeju Lekki. The 2006 National Population Census figures did not include wards in LGAs. However, the 1991 National Population Census contained the number of wards (246) in 20 LGAs. Within the 20 LGAs in the 3 senatorial districts, 113 wards/constituencies were randomly selected from 246 wards, according to urban and semiurban stratification. Then, in each ward, we randomly selected 24 wards out of 113 wards at 40% by proportional allocation techniques (24), as Lagos State Ministry of Health positions health community workers in those selected wards for health intervention projects.

Fourteen communities were randomly selected from the 24 wards as they fit the criteria of being urban or semiurban. Ten urban communities were randomly selected from Lagos West and Lagos Central senatorial districts (Okekoto, Keke, Alapere, Awodi-Ora, Oko-Oba, Agbarawu-Obadina, Oju-Oto, Epetedo, Iwaya, and Igbobi), while four semiurban communities were randomly selected from Lagos East senatorial district (Etita, Erodo, Ilara, and Ijede) [24].

The estimated number of households in the selected 10 urban and 4 semiurban communities in Lagos State population census figures was 119,452 using the projection formula [25].

(1) $$\begin{array}{c} \left(P_{n}=E\left(1\right)+\frac{{\left(GR\right)}^{n}}{100}\right), \end{array}$$

where *E* = 2006 population, GR represents the growth rate of 3.2%, and *n* represents the number of years (1991–2018). While the sampling frame for this study was obtained by factoring in the 2016 projected population of the selected communities by the average household size of 6.5 [24], this gave a total of 18, 377 including both male and female population. The precensus list was used to determine 1,850 households containing women of childbearing age in the selected urban and semiurban areas to avoid bias as well as to ensure an equal chance of selection. A maximum of two respondents was recruited from each household, obtained from a complete list of the 2006 enumeration areas (EAs) of the selected communities. Finally, a total of 850 women of childbearing age, resident in Lagos State for at least 5 years, and aged between 15 and 49 years were purposively and equally selected from the sampled households. Besides, the purposive sampling technique was adopted from studies that have used the technique in the selection of respondents and kidney diseases [26–28].

### 2.3. Data Collection

A pretest structured questionnaire was used to collect information on sociodemographic factors, knowledge of KD and CKD risk factors (independent and dependent), perceived risk of self-reported CKD risk factors, and perception of CKD risk factors. The initial draft of the questionnaire on sociodemographics, knowledge, perceived risk, and perception of CKD risk factors was generated through a literature review of existing public [29–33] and related modified versions of questionnaires [34–40], following discussion with nephrology and social science research experts. The questionnaire was reviewed for content and face validity by medical sociologists (*n* = 2), health demographers (*n* = 3), nephrologists (*n* = 3), and public health practitioners (*n* = 2). Internal consistency of the instrument was verified by the use of Cronbach's alpha (*α*) and was evaluated only between the perceived risk and perception questions, which had a uniform pattern of responses based on the Likert scale as well as the instrument's heterogeneity of responses [41, 42]. The higher the *α* coefficient, the more consistent is the questionnaire in measuring the variable under study [43, 44]. The Cronbach's *α* was set at 0.5 for this current study.

The questionnaire instrument comprises three sections. The first section includes the sociodemographic characteristics of the study respondents. The second section consists of independent risk factors (such as misuse of analgesics (NSAIDs), herbal drinks, and herbal supplements) and dependent risk factors (such as diabetes, hypertension, family history of KD ailments, high cholesterol, and cigarettes smoking). The third section involves the signs and symptoms of CKD ailments. Thus, the independent and dependent risk factors were assessed through self-reporting with clinical proof by the study respondents.

Research assistants were trained for data collection in August 2019. Data were collected in the randomly selected ten urban and four semiurban communities across the three senatorial districts of Lagos state. Copies of the questionnaire were administered both in English and the major local language in the selected study community (the Yoruba ethnic group). Permission to carry out the study in the selected communities was received from opinion and community (Baale) leaders. Data were collected on-site during house-to-house visits that were conducted between 7 am and 12 pm or at the nearest community public ground when respondents did not live far away from this facility, as agreed upon by the study respondents. Data were collected from 850 respondents, and 825 questionnaires were included in the final sample as they had adequate data for analysis. The University of Ibadan Social Sciences and Humanities Research Ethics Committee (SSHEC), Nigeria (UI/SSHEC/14/0003), approved the study.

### 2.4. Definition of Terms

In this study, the definition of terms were mainly focused on the CKD risk factors used in this study and were categorized as independent and dependent risk factors, as adapted from previous studies [9, 10, 45–51]. For instance, the self-reported history of long-term exposure and/or misuse of NSAIDs with clinical proof in the past three months before the study was obtained [9, 10]. Herbal drink (herbal drinks mentioned by respondents were “Alomo” (for libidinal efficacy), “Opa Eyin” (for waist pain), “Wiper Bitter” (for cleanser), and “Bulldozer” (for washing the stomach)) was self-reporting of frequent intake of combination of herbal concoction locally made called “agbo” [45]. Herbal supplements (herbal supplements mentioned by respondents were “Agbo Jedi-Jedi” (for pile), “Ogun Iba” (for malaria), “Ogun Eje” (for blood), and “Dogonyaro”(for antibacterial)) are self-reporting of frequent ingestion of herbal medication as extracts, teas, powders or tablets, or capsules made from plants, fungi, or algae [46]. Diabetes was obtained from the self-reported history, with clinical proof of use of insulin or an oral hypoglycemic agent [47]. The self-reported history of hypertension with clinical proof was defined as the medical history of hypertension and/or the use of antihypertensive medication in the past three months before the study [48]. The family history of kidney disease was self-reporting of an inherited form of the disease running in the families or being passed down through one or more families' generations [49]. The self-reported history of high cholesterol with clinical proof was defined as abnormal raised levels of cholesterol that trigger the inflammation of the kidney organ [50]. Self-reporting of excessive smoking of cigarettes/tobacco was defined as smoking more than one cigar pack per day [51].

### 2.5. Measures

The outcome and independent variables were obtained from the responses of the respondents. The outcome of interest in this study was self-reported perceived risk/perception and categorized for appropriate interpretation as follows: perceived risk was categorized into low and high and perception was categorized into poor and good. Also, KD knowledge was derived from the following question: “Have you heard about kidney disease?” and respondents' responses was dichotomized into “Yes” or “No.” Responses from kidney disease types were derived from the following questions: “What are the types of kidney disease?” (acute KD only, chronic KD only, and both acute and chronic KD). We grouped the first two categories in poor knowledge of KD and the last one in good knowledge of KD.

The perceived risk was categorized low and high, while perception was dichotomized into poor and good values by series of 40 multiple-choice questions, which were generated from the independent and dependent CKD risk factors. The independent risk factors of CKD are free from external control of other diseases to cause CKD. In this study, the independent risk factors of CKD include misuse of analgesics (NSAIDs), herbal drinks, and herbal supplements. Also, the dependent risk factors of CKD are contingent on other ailments to cause CKD. In this study, the dependent risk factors of CKD include diabetes, hypertension, family history of KD ailments, high cholesterol, and cigarettes smoking. These risk factors were dichotomized as “present” (yes) and “absent” (no), and this was done using the CKD risk prediction model with some modifications [44].

Similarly, the perceived risk and perception of CKD risk factors was categorized as low (<20) and high (≥20). Respondents with high perceived risk or perception were coded “1” and those with low perceived risk or perception were coded “0.” As the main predictor variable, CKD knowledge generated a positive response to a series of knowledge questions generated from previous CKD knowledge surveys and its CKD risk factors [28–39]. CKD's knowledge and beliefs were assessed by asking about the types of KD (acute and chronic KD), how people get kidney problems, knowledge of CKD risk factors, and CKD's signs/symptoms. Knowledge of respondents was scored as poor or good based on the proportion of the total score [28–30]. The total score was 40, and <20 was poor, while ≥20 was good knowledge.

Equally, in this study, the independent variable include age, education, marital status, religion, income, other sources of income, and ethnic group. Thus, age cohorts were recoded as younger adults [15–29], young adults [30–39], and middle-aged adults [40–49, 52]. Education was recoded as no education, low education (primary and secondary), and high education (tertiary). The variable marital status was coded as single, married, and previously married. The variable religion was coded as Christianity, Islam, and Traditional. The income variable was coded as low and high, and in this study, a low-income variable was indicated by gross household income less than the minimum wage of 30,000 naira (75 United States Dollars) and high income was indicated by gross household income higher than the minimum wage of 30,000 naira (75 United States Dollars), respectively [53]. Equally, other sources of income were coded as yes and no while the ethnic group was recoded as Yoruba and non-Yoruba.

### 2.6. Data Analysis

Statistical analyses were performed with the Statistical Package for Social Sciences (SPSS) version 25. Data were presented as mean ± standard deviation. Categorical variables were computed using frequencies, and percentages and graphs were used to determine knowledge of CKD risk factors, perceived risk, and perception of CKD risk factors. On the other hand, the bivariate analysis was employed to show the comparisons of proportions of respondents to determine respondents' knowledge of CKD risk factors by rural and urban areas using Pearson's chi-square test. Regarding the multivariate analysis, binary and multinomial logistic regressions were employed to estimate the behavioural, demographic factors, and independent and dependent risk factors of CKD. The binary logistic regression analysis was carried out to estimate the behavioural and demographic factors associated with respondents' increased knowledge of CKD risk factors. Similarly, multinomial logistic regression was employed to estimate the perception and perceived risk associated with knowledge of CKD risk factors (independent and dependent). Other variables such as age cohorts, education, and income (demographic factors) were also included in the multivariate analysis. The level of significance was set at *p* ≤ 0.05.

## 3. Results

### 3.1. Demographics

A total of 850 respondents participated in the study, but 25 were not eligible for analysis due to incomplete data. The data of 825 women aged 15–49 years with mean age of 33.5 ± 11.5 years (urban mean age: 30.2 ± 10.9 years; semiurban mean age: 43.7 ± 6.3 years) with a range of 15–49 years were analysed.  Table 1 provides the sociodemographic characteristics of the respondents. Compared to the semiurban communities, more respondents were younger (less than 30 years old), more educated, low income, and more of Christianity were found in the urban areas, as given in Table 1.

### 3.2. Distribution of Respondents' Level of Knowledge of CKD Risk Factors

**Figure 2:**
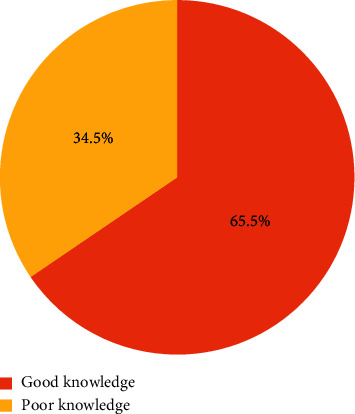
Level of knowledge of kidney disease risk factors among respondents.

In this section, the level of knowledge of CKD risk factors among the respondents were determined and CKD risk factors were categorized as independent and dependent. Thus, Figure 2 shows that 540 (65.5%) of the respondents had a good knowledge of CKD risk factors as against 285 (34.5%) of them with poor knowledge of CKD of risk factors (Figure 2).

### 3.3. Distribution of Respondents' Level of Knowledge of CKD Risk Factors by Age

**Figure 3:**
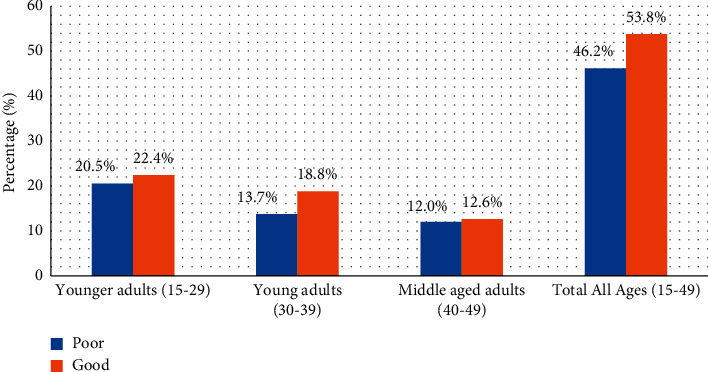
Level of knowledge of CKD risk factors among participants by the age group.

Similarly, Figure 3 shows that younger adult respondents (15–29 years) had better knowledge (22.4%) of CKD combined risk factors compared to young adults (30–39 years) and middle-aged adults (40–49 years), respectively (Figure 3).

### 3.4. Association between Respondents' Knowledge of Kidney Disease and CKD Risk Factors

This section deals with explanation of association of the general of knowledge of KD and CKD risk factors. The analysis was done by cross tabulating selected independent variables with the respondents' CKD risk factors (independent and dependent) by location (Table 2). The findings showed evidence of significant associations between knowledge of KD by urban/semiurban residence. Knowledge of KD is a factor that showed a significant association with a place of residence (*p* < 0.05). Surprisingly, higher proportion of respondents from urban (68.5%) and semiurban (63.7%) place of residence reported both acute and chronic as type of KD (*p* < 0.05). Similarly, majority of the respondents reported that male gender is more susceptible to CKD (urban: 66.5%; semiurban: 54.8%) and showed a significant association with respondents' knowledge of CKD by place of residence (*p* < 0.05) (Table 2). However, the proportion of respondents who mentioned the misuse of analgesic (NSAIDs) is higher in the urban (22.5%) residence compared to those residents in the semiurban (11.9%) areas. Herbal drinks showed a significant association with respondents' knowledge of CKD risk factors (*p* = 0.05). The use of herbal supplements is found to be higher among semiurban respondents (34.8%) compared to those residing in the urban (23.7%) areas (*p* < 0.05) (Table 2). Equally, out of the five variables included in the analysis for dependent risk factors for CKD, only hypertension (urban: 9.0%; semiurban: 14.1%) and family history of CKD ailments (urban and semiurban: 8.1%) showed a significant association as a dependent risk factor for CKD that will influence respondents' knowledge of CKD (*p* < 0.05) (Table 2).

### 3.5. Self-Reported Independent and Dependent Risk Factors among the Respondents with CKD Knowledge

Table 3 provides the logistic regression predicting the respondents' self-reported independent and dependent CKD risk factors, respectively. Regarding independent CKD risk factors, the odds of misuse of analgesics (NSAIDs) increased significantly by 12% among respondents with high level of knowledge. Also, respondents with high level of knowledge who consumed herbal drinks (OR = 2.302, CI = 0.975–3.240; *p* < 0.05) and herbal supplements (OR = 1.372, CI = 0.881–2.139; *p* = 0.05) were 23% and 14% times as likely as those in reference category (RC) who do not consume herbal drinks and supplements (Table 3). Similarly, respondents with knowledge of CKD reported higher odds of predicting hypertension as dependent risk factors for CKD (OR = 2.135, CI = 1.106–4.119; *p* < 0.05). The odds of reporting the family history of KD ailments as dependent risk factors for CKD increased significantly by 13% among respondents with knowledge of CKD (OR = 1.302, CI = 0.215–1.742; *p* = 0.05). Also, respondents with CKD knowledge significantly predicted higher odds (14%) of high cholesterol as dependent risk factors of CKD (OR = 1.441, CI = 0.220–1.824; *p* = 0.05) (Table 3).

### 3.6. Respondents' Level of Perceived Risk towards CKD Risk Factors

**Figure 4:**
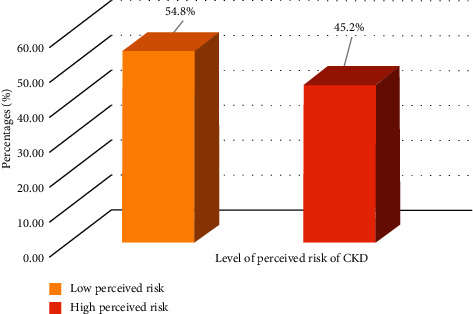
Level of perceived risk towards CKD ailments among respondents.

In terms of level of perceived risk towards CKD risk factors, as shown in Figure 4, majority of the respondents had low perceived risk (54.8%) towards CKD risk factors despite their increased level of knowledge of CKD risk factors (independent and dependent) (Figure 4).

### 3.7. Perceived Risk of Chronic Kidney Disease Risk Factors among Respondents

Table 4 provides the composite measure of factors that influences the perceived risk of respondents towards CKD risk factors. These measures of factors include selected behavioural and demographic responses and independent and dependent risk factors. Behavioural and demographic factors were rooted in respondents' beliefs and knowledge of how variety of general conditions can result into KD ailments. Thus, this study' findings showed that women with low perceived risk (OR = 0.12, CI: 0.42–1.32; *p* > 0.05) were less likely to indicate that they can never develop CKD compared to those with high perceived risk (Table 4).

Regarding age cohorts, young adults (30–39 years) and middle-aged adults (40–49 years) women are 0.25 and 0.62 times less likely to report that CKD cannot occur among age cohorts of 30 years and above as compared to those in the reference category. As regards independent risk factors, women who engaged in consumption of herbal drinks and misuse of analgesic (NSAIDs) were 0.28 and 0.46 times less likely to report their perception towards independent CKD risk factors compared to those in the reference category. Similarly, dependent risk factors such as hypertension and family history of KD ailments are significant factors predicting decreased likelihood of perceived risk towards dependent CKD risk factors among women compared to those in the reference category (Table 4).

### 3.8. Respondents' Level of Perception of Risk Factors for CKD

**Figure 5:**
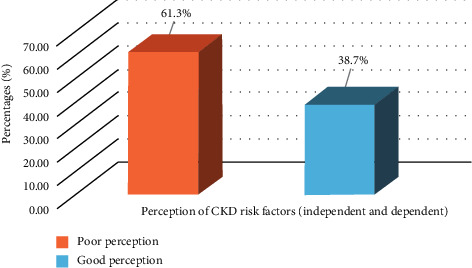
Level of perception of risk factors among respondents.

Figure 5 shows an overall level of respondents' perception of risk factors CKD and risk factors are grouped as dependent and independent. The data as shown in Figure 1 show that a majority of the respondents had poor perception (61.3%) of CKD risk factors and 38.7% of the respondents had good perception of CKD risk factors, as shown in Figure 5.

### 3.9. Predictors of Respondents' Knowledge and Perceived Risk Associated with CKD

This section presents the multivariate analysis of variables.  Table 5 provides the binary and multinomial logistic regression analyses of the predictors of respondents' knowledge and perceived risk associated with CKD risk factors. Thus, Table 5 is broken down into 5A, 5B, and 5C. Section 5A provides the binary regression analysis of demographic factors associated with respondents' increased knowledge; while section 5B provides the multinomial logistic regression analysis of perceived risk associated with knowledge of CKD independent risk factors; and section C provides the multinomial logistic regression analysis of perceived risk associated with knowledge of CKD independent risk factors. Sociodemographic factors such as younger aged adults (15–29 years), high education, and high income were 1.2 times, 3.39 times, and 2.44 times as likely as those in reference category to report that demographic factors significantly predicted respondents' increased knowledge (Table 5, Section A). Similarly, independent factors such as misuse of analgesics (NASAIDs), herbal drink, and herbal supplement were 1.96 times, 2.01 times, and 1.23 times as likely as those who predicted perceived risk associated with knowledge of CKD independent factors in the reference category (Table 5, Section B). Equally, the odds of reporting hypertension (OR = 1.95, CI: 1.32–3.20; *p* < 0.05) and family history of KD ailments (OR = 1.15, CI: 0.32–1.46, *ρ* < 0.05) as dependent risk factors increased significantly by 20% and 12% among respondents with “Yes” responses compared to those who said “No” as responses (Table 5, Section C).

## 4. Discussion

The study had focused on the knowledge, perceived risk, and perception of CKD risk factors using a cross-sectional primary data of representative sample of women of childbearing age (15–49 years) in Lagos State, Nigeria. To our knowledge, this is the study that examines the knowledge, perceived risk, and perception of CKD risk factors among women of childbearing age in Lagos State, Nigeria. Knowledge of CKD risk factors was measured from the information on seven indicative questions of knowledge level of CKD risk factors to prevent CKD ailments and promote prevention measures of independent and dependent CKD risk factors. Evidence from the study shows that majority of women have good knowledge of KD and CKD risk factors, respectively. This finding is consistent with other studies that have documented good knowledge of KD and CKD risk factors among Nigerian women [3, 4, 10]. Good knowledge of KD and CKD risk factors as documented in this study and other past studies show intensive enlightenment in the health education programmes on CKD risk factors in Nigeria. When disaggregated by age, the study shows huge knowledge gap, with higher proportion of younger adult women (15–29 years) reported good knowledge of KD and CKD risk factors compared to other age cohorts (young adult (30–39 years) and middle-aged adult women (40–49 years) [6, 34]. The present finding underscores knowledge imbalance of CKD risk factors by women's age. This finding is consistent with Oluyombo et al. [28] and Akokuwebe et al. [10] who reported in their studies that younger adults (15–29 years) had a better knowledge of CKD risk factors than young and middle-aged adult women. However, Oluyombo et al. (2013) and Roomizadeh et al. (2014) in a different study in Western Africa and South Asian reported that older women have higher knowledge of CKD risk factors, but none of these studies made recourse to factors accounting for the knowledge gap by age. Also, the findings from the bivariate analysis showed that in-depth knowledge of CKD risk factors was significantly associated with general knowledge of KD (self-awareness of KD, types of KD, gender susceptibility to CKD, and residing location—urban and rural). Evidence of urban women was found to have a better knowledge of CKD risk factors compared to rural women. This finding is consistent with other studies that reported that urban women have better knowledge of CKD risk factors [4, 10]. Consistent with existing studies, knowledge is influenced by education and age, and the better educated a woman is, the more likely she was to have more general knowledge of CKD risk factors; although older a woman is, the lower her level of knowledge as reported in previous studies [28, 30]. This finding recaps that older women should be targeted for regular and comprehensive health education, as limited health literacy might encourage misconceptions, “denial of health reality” of life-threatening disease, delayed diagnosis, poor lifestyle modifications, and increased CKD burden. Adequate knowledge and awareness of health tips on CKD risk factors will boost women in adopting better lifestyle modifications and management of CKD ailments, if it exists [9, 28]. Therefore, early identification and treatment of CKD will reduce the rate of progression, as well as the burden of the disease complications.

The study finding shows the majority of respondents' self-report account of misuse of analgesics (NSAIDs), herbal drinks, and herbal supplements as independent CKD risk factors, while hypertension, family history of KD ailments, and high cholesterol as dependent CKD risk factors. Thus, NSAIDs are mostly used in reducing inflammation and as pain relievers, and some of the NSAID mentioned by the respondents include ibuprofen and Tylenol (such as acetaminophen, Tempra, Panadol, or paracetamol). Similarly, most of the respondents have strong confidence that herbal drinks (herbal drinks mentioned by respondents were “Alomo” (for libidinal efficacy), “Opa Eyin” (for waist pain), “Wiper Bitter” (for cleanser), and “Bulldozer” (for washing the stomach)) and herbal supplements (herbal supplement mentioned by the respondents include “Agbo Jedi-Jedi”(for piles), “Ogun Iba” (for malaria), “Ogun Eje” (for blood), and “Dogon Yaro” (for antibacterial)) are better remedies for other chronic ailments such as diabetes, hypertension, and also to cure any kidney ailments. These NSAIDs and herbal drinks mentioned in this study have been in consistent with existing studies [54–57]. Evidence of self-reported independent risk factors significantly predicted the likelihood of women engaging in their recounted CKD risk factors. This assertion was documented in the works of Oluyombo et al. [31] and Roomizadeh et al. [30]. Similarly, concerning dependent risk factors, respondents' indication of hypertension, family history of KD ailments, and high cholesterol significantly predicted the increased odds of women having these risk factors, without CKD diagnosis. This further support previous findings of the aforementioned dependent CKD risk factors that are prevalent among women, with increasing age [6, 10, 28], and such women with combined independent and dependent CKD risk factors are likely to be susceptible to CKD ailment [48–50]. This finding agrees with other previous studies conducted among African-American women of the United States of America [58–61]. Surprisingly, risk factors are influenced by natural course of body hormones which are complex and heterogeneous and have been identified to trigger disease development and progression in different stages of any illness. These findings can construe that risk factors may contribute to KD ailments, as abnormal levels of these aforementioned dependent factors can pose direct and specific risks to health [62, 63], as they are critically examined along with aspects of biological characteristics (genes), behavioural factors (lifestyle and health beliefs), and social conditions (cultural influences, family relationships, and social support). This is key in the aspects of epidemiology of health, public health, and in health demography, as they are key personal health risk factors which have an impact on health matters. Some of these factors could be changed, while others cannot be changed. It is especially important for individuals at increased risk of CKD to have prior information [63–65].

The study revealed that early identification of CKD risk factors and treatment of its ailments will reduce the rate of progression, as well as the burden of the disease complications, thus enhancing the quality of lives, especially at the reproductive phases of women's lives [11, 12]. A study conducted by Piccoli et al. [3] and Starring et al. [4] revealed that individuals had inadequate knowledge despite efforts medical team put in creating awareness, and this has shown that physician' present education strategies do not meet the unmet needs of women who are exposed to CKD risk factors and unknowingly to the ailments. This finding may reflect a major gap in community-health workers communication, as it may be that those women may not be receiving adequate health education on CKD or that women do not understand the health information that was provided on CKD and its risk factors. The present finding emphasizes imbalance of knowledge between women of reproductive age and health professionals. This finding is consistent with Luyckx et al. [66] who reported in their study that community health workers' understanding of women's level of health literacy is crucial to improving the quality of their communication experience with healthcare professionals. This finding demonstrates the need for a change of education strategies and the provision of additional resources and healthcare personnel to support women education in CKD risk factors and its associated ailments if modification of CKD risk factors were not adopted by women. Thus, the identification of factors predisposing an individual to CKD is essential in terms of personal and community health, as some risk factors can be modified and can prevent or slow down its progression to end-stage renal disease (ESRD) [7, 11, 12].

Overall, the findings show that majority of the respondents had low perceived risk (54.8%) towards CKD risk factors (independent and dependent). While the percentage of women who view themselves at increased risk for CKD was relatively low among the young and middle-aged adults compared to those in the younger adults' age cohorts. However, those with self-reported independent CKD risk factors such as herbal drinks and misuse of analgesics (NSAIDs) were less likely to view them themselves at increased risk for renal impairment compared to those without the use of herbal drinks and misuse of analgesics (NSAIDs). Furthermore, women with self-reported hypertension and family history of KD ailments were less likely to consider themselves at increased risk for the disease compared to those without hypertension and family history of KD ailments. In fact, diabetes, high cholesterol, and cigarette were not found as predictors of increased CKD risk in the multivariate binary regression model. While the underrecognition of kidney disease risk is studied, yet it is not unprecedented. One possible explanation for the low perceived risk for CKD may be due to the “silent nature” of early kidney disease expression [14–16]. While the consequences of the independent CKD risk factors on the kidney organ are well known, glomerular filtration rate and its implications for kidney disease is less part of the cultural vernacular. The complications of the combined independent and dependent CKD risk factors are more acute, whereas symptoms of kidney disease are often silent, and the slow progression of the disease can make it unnoticeable to the individual [28–34]. The link between independent risk factors and CKD seems to be more known among women than the association between dependent risk factors and CKD. This finding is consistent with the health belief model and from other studies, which suggest that it is understandable why the risk factors with a lack of acute symptomatology would not result in an increased perceived risk and susceptibility for CKD in our study [67–70].

As expected and consistent with other studies, women's poor perception could be linked to low perceived risk towards CKD risk factors [10, 18], as the study findings reported that most of them showed poor perception (61.3%) of CKD risk factors, even though the potential risks of CKD in self-reported CKD risk factors were obvious. As a result of this, a majority of the respondents are unlikely to engage in precautionary measures in preventing kidney ailments. Hence, perceived risk and perception may be a strong motivating factor for behavioural change, ensuing a perceived control over actions and embracing behavioural modifications among “high risk” population of women with prevalent CKD risk factors [65, 71]. Women with poor perception towards any chronic disease might be connected with either absence or inadequate knowledge of the implications and burden of the disease. Unfortunately, this has become a great concern to health workers, as women seem to believe that they are invincible and invulnerable to ailments related to the kidney organ. Thus, poor perception of CKD risk factors can place women at risk of catastrophic health expenditure, which poses high opportunity costs for health systems. In Nigeria, CKD prevention is highly cost-effective in improving early diagnosis and treatment, but requires a multisectoral holistic approach that will design interventions that will reduce the need for high-cost care [8, 10, 28]. Health literacy programmes should be aimed at changing misconceptions, thereby improving positive attitudes and enhancing better-adapted approaches in dealing with chronic illnesses such as CKD.

In the multivariate analysis, demographic factors such as younger aged adults (15–29 years), high education, and high income significantly predicted the likelihood of increased knowledge of CKD risk factors among respondents. As evidence in the study, independent risk factors such as misuse of analgesics (NASAIDs), herbal drinks, and herbal supplements were more likely to predict the perceived risk associated with knowledge of CKD risk factors among women. While, dependent risk factors such as hypertension and family history of KD ailments significantly predicted the likelihood of perceived risk associated with knowledge of CKD risk factors among the respondents (*p* = 0.03 < 0.05; *p* = 0.02 < 0.05). This is similar to the report of other studies [3, 10]. Therefore, emphasis should be placed on health literacy programmes geared towards improving women's responsiveness and understanding of CKD risk factors, as this will educate them on various risk factors and their health implications. Women should have adequate knowledge on demographic, independent, and dependent CKD risk factors which may directly or indirectly influence CKD risk factors and its ailments. From a health demography perspective, demographic factors are key in investigating specific features of individual health risk factors to create interventions that will take cognizance of social and demographic determinants of health, along with the underlying independent and dependent CKD risk factors [5, 6, 9, 10]. Health promotion and disease prevention efforts will likely play a larger role in healthcare services, thus creating awareness of CKD and risk factors involved.

Therefore, the study recommends that regular health check-ups are known to be effective in detecting and treating chronic diseases at an early stage. The findings of this study showed that women were less likely to perceive the usefulness of regular health assessments as an effective CKD preventive measure. This is due to a low perceived risk and poor perception of the association between CKD risk factors and its ailments. This indication requires increased efforts to reach out to women to educate them on the benefits of regular medical check-ups and screening. In addition, some myths contribute to CKD neglect in women. One such is the persistent interpretation of health issues related to women through their anatomical reproductive capacity. As a result of this, frequent misperception occurs in CKD risk factors associated with males, with CKD perceived as a disease of men. Women diagnosed with CKD are always identified among women with lifestyle choices resident in high-income countries [2, 3, 72]. Given this, targeted policy programmes and health interventions among women should be highlighted. These will help to meet the specific needs and context of women concerning noncommunicable diseases such as CKD.

### 4.1. Policy Recommendations, Study Limitations, and Future Research Ideas

Knowledge of CKD risk factors was quite high among younger adult women aged 15–29 years, and with their self-reported CKD risk factors, their perceived risk and perception towards CKD risk were generally low. Younger adult women's predispositions to CKD risk factors were 22% greater than young adult women (19%) and middle-aged adult women (13%). Most clinical studies only provided a gender-specific prevalence of CKD, which was either found in females or males. Based on the outcome of this study, we recommend that women's health should be made a priority in health policies. This will improve well-structured health programmes that will address the health needs of women, especially in the areas of noncommunicable diseases. Urgent attention needs to be paid to preventive measures and intervention to slow women's involvement in unhealthy lifestyles. Community health workers should be motivated to carry out health awareness programmes to discuss lifestyle and biomedical factors that predispose women to CKD development during childbearing age. Screening programmes and treatment facilities should be provided during awareness talks, especially in communities with high-risk populations for CKD. Women who are diagnosed with CKD before or during pregnancy should be given special medical attention. Also, the government should make special medical insurance that will cater to such women with CKD needs. Most of all, women in grassroots communities should be targeted for CKD-related and other preventive health programmes. This study had several strengths and limitations. The strength of our study was a combination of multistage, stratified, and equal sampling methods. Besides, to a large extent, there was good coverage of urban and semiurban communities where the study was carried out. The sample of the respondents who participated in the survey is large and allows for a robust analysis of the research problem. The study found a connection between CKD knowledge, perceived risk, and perception of risk factors among women of childbearing age in Lagos State, Nigeria. Based on the authors' knowledge, this study is the first nonclinical study of its kind, using a health demography approach to address women-related CKD as a subject matter, which has a reflective effect in urging women to know their medical history in their childbearing phases of life. One of the few shortcomings of this study is the adoption of a cross-sectional research design, which seems to provide a limited view of respondents' risk factors over time. In other words, the study did not include a medical diagnosis of the diseases, while detailed self-reporting was adopted for those diagnosed with other common chronic diseases. The knowledge and risk perception issues that the researchers looked at were based on social matters and measured with a psychological scale of measurements [9, 24, 32]. The CKD knowledge and its risk factors were self-reported, thereby making the concern of recall bias of importance. The determination of independent and dependent CKD risk factors did not involve the length of time they were involved in such risk factors and such information was not obtained. Because of the cross-sectional nature of this study, we suggest that longitudinal studies on the causation between perceived risk of CKD risk factors and ailment progression should be considered in future health as well as demography surveillance sites or surveys. Although knowledge and medically scientific analyses of the causes of CKD risk factors within a population have significantly been improved in recent times, the specific opportunities for future research should focus on key awareness of health disparities, which occur in developing countries, particularly in Africa. From previous studies, women are seen to not to be documented with CKD risk factors and treatment [3, 10]. Thus, women and CKD remain one of the most crucial research areas that are continually being neglected. Research intervention and policy frameworks should be provided to stimulate thought and interests for future research among the CKD research community and health demographers. Consequently, this will improve the understanding of specific CKD policy interventions that will maximize health benefits and minimize risks among women populations.

## 5. Conclusion

Knowledge of CKD risk factors are quite high among women of childbearing age in Nigeria. Many of the respondents among the young and middle-aged cohorts had a low-risk perception towards CKD, with more prevalent independent and dependent CKD risk factors, which have severe health implications. Despite the women's knowledge of CKD risk factors, it could be deduced that women do not have adequate information on the adverse effects of these risk factors on the kidney organ. It is pertinent to infer that women do not perceive that they are prone to CKD and its associated ailments. Therefore, women must be provided with adequate knowledge and clarification that a female has a higher chance of being at risk of CKD if they are engaged in certain CKD risk factors. This recommendation could be achieved through sensitization, timely diagnosis, and proper follow-up of women during public enlightenment programmes. To pose a positive impact on CKD reduction among generations of women, CKD advocacy programmes should be implemented across all levels of government. Furthermore, health demographers, sociologists, and community health workers should strategize and design health intervention programmes that will assist and accommodate “high risk” women of childbearing age in CKD-related health facilities with other dependent chronic ailments. Women in the grassroots communities should be targeted for CKD screening programmes and other chronic ailments as they do not have full access to health facilities in these communities. Besides, the Nigerian government should intensify a public CKD health policy that supports behavioural interventions with the inclusion of the female population in the policy intervention, regarding their overall health.

## Figures and Tables

**Table 1:** Characteristics of study respondents.

Characteristics	Urban, *n* (%), *n* = 624	Semiurban, *n* (%), *n* = 201	Total, *n* (%), *N* = 825
Total mean age	30.2 ± 10.9	43.7 ± 6.3	33.5 ± 11.5
Age			
Younger adults (15–29 years)	280 (44.9)	74 (36.8)	354 (42.9)
Young adults (30–39 years)	195 (31.3)	73 (36.3)	268 (32.5)
Middle-aged adults (40–49 years)	149 (23.9)	54 (26.9)	203 (24.6)

Education			
No education	18 (2.9)	190 (94.5)	208 (25.2)
Low	44 (7.1)	9 (4.5)	53 (6.4)
High education	562 (90.1)	2 (0.1)	564 (68.4)

Marital status			
Single	352 (56.4)	89 (44.2)	441 (53.5)
Married	230 (36.9)	96 (47.8)	326 (39.5)
Previously married	42 (6.7)	16 (8.0)	58 (7.0)

Religion			
Christianity	469 (75.2)	134 (66.7)	603 (73.1)
Islam	143 (22.9)	50 (24.9)	193 (23.4)
Traditional	12 (1.9)	17 (8.4)	29 (3.5)

Income			
Low	381 (61.1)	141 (70.1)	522 (63.3)
High	243 (38.9)	60 (29.9)	303 (36.7)

Other sources of income			
Yes	188 (30.1)	87 (43.3)	275 (33.3)
No	436 (69.9)	114 (56.7)	550 (66.67)

Ethnic group			
Yoruba	323 (51.8)	109 (54.2)	432 (52.4)
Non-Yoruba	301 (48.2)	92 (45.8)	393 (47.6)

Source: fieldwork, 2019; ^*∗*^*p* < 0.05, significant. Data are expressed as mean ± standard deviation, number, percentage, and chi-square.

**Table 2:** Bivariate analysis of respondents' knowledge of KD and chronic kidney disease risk factors by location (*N* *=* 825).

General questions	Urban, *n* (%), *n* = 624	Semiurban, *n* (%), *n* = 201	*χ* ^2^	*P*
Have you heard about kidney disease?				
Yes	409 (65.5%)	135 (67.2%)	3.430	0.02^*∗*^
No	215 (34.5%)	66 (32.8%)		
Questions	Urban, *n* (%), *n* = 409	Semiurban, *n* (%), *n* = 135	*χ* ^2^	*P*
Where is the kidney located?				
Close to the spine	74 (18.1%)	39 (28.9%)	8.393	0.14
Close to the abdominal cavity	56 (13.7%)	19 (14.1%)		
Close to the liver	120 (29.3%)	32 (23.7%)		
I do not know	159 (38.9%)	45 (33.3%)		
What are the types of kidney disease?				
Acute (AKD)	31 (7.6%)	12 (8.9%)	28.381	0.00^*∗*^
Chronic (CKD)	98 (23.9%)	37 (27.4%)		
Both acute and chronic	280 (68.5%)	86 (63.7%)		
Which of the genders are more susceptible to CKD?				
Male	272 (66.5%)	74 (54.8%)	10.997	0.01^*∗*^
Female	85 (20.8%)	36 (26.7%)		
Both male and female	52 (12.7%)	25 (18.5%)		
Independent risk factors of CKD				
Misuse of analgesics (NSAIDs) (NSAIDs are nonsteroidal anti-inflammatory drugs that are used to reduce inflammation and relieve pain such as ibuprofen, aspirin, and Tylenol (acetaminophen, Tempra, Panadol, or paracetamol))				
Yes	92 (22.5%)	16 (11.9%)	8.027	0.02^*∗*^
No	02 (0.5%)	00 (0.0%)		
Herbal drinks				
Yes	153 (37.4%)	54 (40.0%)	0.289	0.05^*∗*^
No	256 (62.6%)	81 (60.0%)		
Herbal supplements				
Yes	97 (23.7%)	47 (34.8%)	6.423	0.01^*∗*^
No	312 (76.3%)	88 (65.2%)		
Dependent risk factors of CKD				
Diabetes				
Yes	31 (7.6%)	08 (5.9%)	0.417	0.518
No	378 (92.4%)	127 (94.1%)		
Hypertension				
Yes	37 (9.0%)	19 (14.1%)	2.778	0.02^*∗*^
No	372 (91.0%)	116 (85.9%)		
Family history of CKD ailments				
Yes	33 (8.1%)	11 (8.1%)	2.447	0.04^*∗*^
No	376 (91.9%)	124 (91.9%)		
High cholesterol				
Yes	05 (1.2%)	00 (0.0%)	1.666	0.197
No	404 (98.8%)	135 (100.0%)		
Cigarette smokers				
Yes	03 (0.7%)	02 (1.5%)	1.675	0.642
No	406 (99.3%)	133 (98.5%)		
Signs and symptoms of CKD ailments				
Bloody and frothy urine				
Yes	200 (48.9%)	50 (37.0%)	2.163	0.02
No	209 (51.1%)	85 (63.0%)		
Body swelling				
Yes	24 (5.9%)	36 (26.7%)	6.123	0.345
No	385 (94.1%)	99 (73.3%)		
Loss of appetite				
Yes	45 (11.0%)	28 (20.7%)	2.416	0.072
No	364 (89.0%)	107 (79.3%)		

Source: fieldwork, 2019; ^*∗*^*p* < 0.05, significant. Data are expressed as number, percentage, and chi-square.

**Table 3:** Respondents' self-reported risk factors for CKD, *N* *=* 396.

Independent risk factors	Yes, *n* (%)	No, *n* (%)	Total, *N* (%)	OR (95% CI)	*P*
Misuse of analgesics (NSAIDS)	80 (20.2)	316 (79.8)	396 (100)	1.201 (0.793–1.820)	0.00^*∗*^
Herbal drinks	137 (34.6)	259 (65.4)	396 (100)	2.302 (0.975–3.240)	0.00^*∗*^
Herbal supplements	108 (27.3)	288 (72.7)	396 (100)	1.372 (0.881–2.139)	0.05^*∗*^
Dependent risk factors	Yes, *n* (%)	No, *n* (%)	Total, *N* (%)	OR (95% CI)	*P*
Diabetes	26 (6.6)	370 (93.4)	396 (100)	0.882 (0.394–1.971)	0.76
Hypertension	42 (10.6)	354 (89.4)	396 (100)	2.135 (1.106–4.119)	0.02^*∗*^
Family history of KD ailments	36 (9.1)	360 (90.9)	396 (100)	1.302 (0.215–1.742)	0.05^*∗*^
High cholesterol	03 (0.8)	393 (99.2)	396 (100)	1.441 (0.220–1.824)	0.05^*∗*^
Cigarette smoking	22 (5.6)	374 (94.4)	396 (100)	0.212 (0.310–2.130)	0.58

Source: fieldwork, 2019; ^*∗*^*p* < 0.05 significant; reference category (RC), no response. Data are expressed as number, percentage, and OR.

**Table 4:** Self-reported perceived risk of factors influencing behavioural, demographic, and risk factors for CKD among respondents.

Perceived risk of behavioural and demographic factors	Agree, *n* (%)	Disagree, *n* (%)	Total, *N* (%)	OR (95% CI)	*P*
I can never develop CKD (perceived risk)					
High perceived risk (RC)				RC	
Low perceived risk	234 (36.0%)	416 (64.0%)	650 (100)	0.12 (0.42–1.32)	0.310
CKD does not occur among the following age cohorts:					
Younger adults (15-29 years) (RC)				RC	
Young adults (30–39 years)	241 (37.1%)	409 (62.9%)	650 (100)	0.25 (0.12–1.87)	0.00^*∗*^
Middle-aged adults (40–49 years)	231 (35.5%)	419 (64.5%)	650 (100)	0.62 (0.52–1.68)	0.00^*∗*^
Perceived risk of independent risk factors of CKD	Agree, *n* (%)	Disagree, *n* (%)	Total, *N* (%)	OR (95% CI)	*P*
Independent CKD risk factors					
Herbal supplements (RC)				RC	
Herbal drinks	204 (31.4%)	446 (68.6%)	650 (100)	0.28 (0.72–1.32)	0.00^*∗*^
Misuse of analgesic (NSAIDs)	216 (33.2%)	434 (66.8%)	650 (100)	0.46 (0.49–1.57)	0.00^*∗*^
Perceived risk of dependent risk factors of CKD	Agree, *n* (%)	Disagree, *n* (%)	Total, *N* (%)	OR (95% CI)	*P*
Diabetes					
No (RC)	195 (30.0%)	455 (70.0)	650 (100)	RC	0.14
Yes				0.48 (0.73–1.65)	
Hypertension					
No (RC)	202 (31.1%)	448 (68.9%)	650 (100)	RC	0.04^*∗*^
Yes				0.73 (0.281–1.96)	
Family history of kidney disease ailments					
No (RC)	222 (34.2%)	428 (65.8%)	650 (100)	RC	0.05^*∗*^
Yes				0.64 (0.43–1.84)	
High cholesterol					
No (RC)	203 (31.2%)	447 (68.8%)	650 (100)	RC	0.23
Yes				0.62 (0.44–0.98)	
Cigarette smoking					
No (RC)	75 (11.5%)	575 (8.5%)	650 (100)	RC	0.08
Yes				0.72 (0.79–1.99)	

Source: fieldwork, 2019; ^*∗*^*p* < 0.05, significant. Data are expressed as number, percentage, and odds ratio.

**Table 5:** Binary and multinomial logistic regression analyses of predictors likely to influence knowledge and perceived/perception of CKD risk factors among respondents.

Section A. Binary logistic regression analysis of demographic factors associated with respondents' increased knowledge				
Demographic factors	*β*	SE	Odds ratio (OR)	*P*
Younger aged adults (15–29 years)				
No (RC)			1	
Yes	0.249	0.182	1.42 (0.68–1.65)	0.02^*∗*^
High education				
No (RC)			1	
Yes	0.418	0.242	3.39 (1.01–3.65)	0.00^*∗*^
High income				
No (RC)			1	
Yes	0.261	0.148	2.44 (1.29–2.80)	0.00^*∗*^
Section B. Multinomial logistic regression analysis of perceived risk associated with knowledge of CKD independent risk factors				
Independent risk factors of CKD	*β*	SE	Odds ratio (OR)	*P*
Misuse of analgesics (NASAIDs)				
No (RC)			1	
Yes	0.207	0.479	1.96 (1.32–2.31)	0.00^*∗*^
Herbal drink				
No (RC)			1	
Yes	0.523	0.164	2.01 (1.22–2.32)	0.00^*∗*^
Herbal supplements				
No (RC)			1	
Yes	0.426	0.218	1.23 (0.82–1.95)	0.04^*∗*^
Section C. Multinomial logistic regression analysis of perceived risk associated with knowledge of CKD dependent risk factors				
Dependent risk factors	*β*	SE	Odds ratio (OR)	*P*
Diabetes				
No (RC)			1	
Yes	0.213	0.564	0.10 (0.23–1.15)	0.20
Hypertension				
No (RC)			1	
Yes	0.847	0.293	1.95 (1.32–3.20)	0.03^*∗*^
Family history of KD ailments				
No (RC)			1	
Yes	0.416	0.137	1.15 (0.32–1.46)	0.02^*∗*^
High cholesterol				
No (RC)			1	
Yes	0.020	0.145	0.54 (0.57–1.23)	0.30
Cigarette smoking				
No (RC)			1	
Yes	0.132	0.236	0.61 (0.30–1.76)	0.12

Source: fieldwork, 2019; ^*∗*^*p* < 0.05, significant. Data are expressed as odds ratio.

## Data Availability

The EXCEL and SPSS dataset format used and/or analysed to support the findings of this study are included within the supplementary information file (additional file 1).

## Abbreviations

KD: Kidney disease
CKD: Chronic kidney disease
OR: Odds ratio
CI: Confidence interval
P: *P* value
*N*: Total sample size
*n*: Total population size
eGFR: Estimated glomerular filtration rate
SPSS: Statistical Package for Social Sciences
BMI: Body mass index
SSHEC: Social Sciences and Humanities Research Ethics Committee
GR: Growth rate
LGAs: Local government areas
NHIS: National Health Insurance Scheme
SLE: Systemic lupus erythematosus
UTIs: Urinary tract infections.
